# Dataset of vocabulary in Uzbek primary education: Extraction and analysis in case of the school corpus

**DOI:** 10.1016/j.dib.2025.111349

**Published:** 2025-02-03

**Authors:** Khabibulla Madatov, Sapura Sattarova, Jernej Vičič

**Affiliations:** a Urgench State University, 14, Kh. Alimdjan str, Urgench city, 220100, Uzbekistan; b University of Primorska, FAMNIT, Glagoljaska 8, 6000 Koper, Slovenia; c Research Centre of the Slovenian Academy of Sciences and Arts, The Fran Ramovš Institute, Novi trg 2, 1000 Ljubljana, Slovenija

**Keywords:** Lemma, Uzbek language, Primary school, Corpus construction, Natural language Processing (NLP), Comparative lemma extraction method

## Abstract

The main goal of this research work is to determine the number of new words that a primary school pupil should know/acquire during each academic year. To accomplish this, we have created two datasets. The first dataset was compiled based on the ``Explanatory Vocabulary of the Uzbek Language'' (EDUL). The second dataset was created from 35 primary school textbooks for grades 1-4 approved by the Ministry of Preschool and School Education of the Republic of Uzbekistan, and it was named the ``Uzbek Primary School Corpus'' (UPSC) by authors. Using the ``Comparative Lemma Extraction Method'' (CLEM) proposed by the authors of the article, a vocabulary for grades 1-4 was created, and the problem of determining the number of new words (disregarding word forms as Uzbek is a morphologically rich language) that primary school pupils should learn each academic year was solved.

Specifications Table

**Table:** 

Subject	Computer Science Applications
Specific subject area	Natural language processing
Type of data	Table
Data collection	The dataset consists of two raw texts: The first dataset was collected from the six-volume book “Explanatory Vocabulary of the Uzbek Language” (EDUL), which describes all the words and their explanations in the Uzbek language. This dataset includes all Uzbek words ordered sequentially in a column. It was compiled manually by computer science teachers and master's pupils in the specialty “Computer Linguistics” at Urgench State University in 2019-2023. The second data was created based on the primary school corpus. This corpus was created using textbooks from the National Book Portal of Uzbekistan (https://kitob.uz/), An alternative to the proposed site is a mobile app available for the Android platform: Maktab darsliklari | Kitoblar. The app is available through the Google Play Store: https://play.google.com/store/apps/details?id=dev.mobile.books&hl=en_US. The tutorials were downloaded in PDF format, pre-processed, cleaned, tokenized, and saved as TXT files using custom Python scripts. A list of URLs has been created for these resources. The results of studying the vocabulary of primary school pupils in grades 1-4 are described in detail in the section “Experimental plan, materials and methods.”
Data source location	Higher educational institution: Urgench State University 14, Kh.Alimdjan str, Urgench city, 220100, Uzbekistan
Data accessibility	Repository name: Zenodo Data identification number: 10.5281/zenodo.13165746 Direct URL to data: 10.5281/zenodo.13165746 **Files:** • **EDUL.txt:** Lemmatized words from EDUL dataset. • **Uzbek Primary School Corpus:** Grade-specific textbooks (Grade1.txt, Grade2.txt, Grade3.txt, Grade4.txt). • **Vocabulary Dataset:** Grade-specific vocabulary (Grade1_Vocabulary.txt, Grade2_ Vocabulary.txt, Grade3_ Vocabulary.txt, Grade4_ Vocabulary.txt). • EDUL.txt • Grade1.txt • Grade2.txt • Grade3.txt • Grade4.txt • Grade1_Vocabulary.txt • Grade2_Vocabulary.txt • Grade3_Vocabulary.txt • Grade4_Vocabulary.txt • new_words_grade_1.txt.txt • new_words_grade_2.txt • new_words_grade_3.txt • new_words_grade_4.txt • unique_words_first_grade.txt • unique_words_fourth_grade.txt • unique_words_second_grade.txt • unique_words_third_grade.txt • frequency_unique_grade_1.txt • frequency_unique_grade_2.txt • frequency_unique_grade_3.txt • frequency_unique_grade_4.txt The data are stored in a simple text format where each line represents a single word.
Related research article	

## 1 Value of the Data

The Dataset of Vocabulary in Uzbek Primary Education obtained as a result of this research is significant for several tasks related to the quality of primary education and the improvement of natural language tools in Uzbek.

• Firstly, it ensures a dataset of new words that pupils of primary education should know every school year
• Pedagogues gain access to a valuable teaching resource.
• Teachers can organize various educational activities using this dataset, such as dictations or exam preparation.
• It supports the development of NLP tools tailored for the low-resource Uzbek language.
• Enhances text processing tools.
• Supports the creation of pedagogical materials, and advances speech recognition and synthesis systems.

This dataset is expected to serve as a highly beneficial resource for the following user groups and applications

• Uzbek language researchers can analyze morphological structures, word formation, and language patterns in primary education using this dataset.
• Pedagogues and curriculum developers can use the vocabulary to create lesson plans and curricula that expand the vocabulary of primary school pupils.
• The dataset provides the basis for the development of intellectually relevant textbooks, workbooks, and additional resources for young learners.
• Helps improve machine translation systems.

## 2 Background

The Uzbek language is a Turkic language spoken by more than 37 million people in Uzbekistan and neighboring Central Asian countries. The main focus of the paper is on automatically extracting primary school vocabulary from Uzbek Primary School textbooks and determining the number of new words that every grade-level primary school pupil should know during a school year.

To achieve this goal, the following steps were taken:

• A vocabulary of all Uzbek words was manually created based on the six-volume “Explanatory Vocabulary of the Uzbek Language” (EDUL) [12].
• 35 primary school textbooks for grades 1-4 were downloaded from https://kitob.uz/ (or android app: Maktab darsliklari | Kitoblar) and this corpus was named “Uzbek Primary School Corpus” (UPSC).
• A dataset of unique words for primary education was created using the Uzbek Primary School Corpus. In Natural Language Processing (NLP), ``unique words'' refer to the distinct words present in a text corpus without considering their frequency. This means each word in the text is counted only once, regardless of how many times it appears.
• Explanatory Vocabulary of the Uzbek Language (EDUL) datasets and unique word datasets were compared using the Comparative Lemma Extraction Method (CLEM). Based on the final results, the number and list of new words that primary school pupils should know in each school year were determined.

These findings enable the enhancement of the quality of primary education through the vocabulary developed in this study:

• To ensure that primary school pupils develop their vocabulary and have accurate writing skills
• Development and implementation of educational programs for primary school.
• Creating educational materials that are suitable for the intellectual abilities of pupils.
• Increase the opportunity for pupils to learn words at home with the participation of parents based on vocabularies created based on textbooks.
• Increasing pupils' ability to independently memorize words by creating digital audio dictionaries and uploading them to special educational platforms
• Increase pupils' vocabulary through regular dictation exercises.

As mentioned above, the main goal of this research work is to create a measure that ensures that the younger generation, the future of our society, develops a quality vocabulary in primary school. Because primary school pupils who do not have a sufficient level of vocabulary will not find their place in society in the future. Therefore, there should be a measure of whether primary pupils have age-appropriate vocabulary. Until now, there has been no dataset or measurement of how many new words a pupil at each level of primary school should know during each school year. Based on the results of this research, a final dataset is presented that details how many words, and how many of them are new words, a primary school pupil should learn in each academic year.

## 3 Data Description

Although the Uzbek language is a low-resourced language belonging to the Turkic family, considerable work is being done in the field of NLP based on the School Corpus [1,2,3,4]. The new vocabulary dataset used in this article was also created from the School Corpus.

Table 1 shows the list of raw texts and files used in creating the primary school vocabulary. The following dataset was created for developing a vocabulary tailored to grades 1-4 of Uzbek primary schools:

1. **Initially, the ``Explanatory Dictionary of the Uzbek Language'' (EDUL**) was converted to text format and saved in text format. This data set, which covers the vocabulary of all the words of the Uzbek language, is stored in the form of a list of 29,190 words.
2. **Uzbek Primary School Corpus (Uzbek Primary School Corpus):** This corpus is based on 35 primary school textbooks, and it contains a total of 208,204 tokens (see Table 2 for comparison). Tokens to mean the total number of running words.

**Table 2:** Names of files and total number of tokens in the Uzbek primary school corpus dataset.

File name	# of entries
Grade1.txt	24,105
Grade2.txt	56,645
Grade3.txt	90,250
Grade4.txt	109,204
3. **The dataset of unique words created based on the Uzbek Primary School Corpus**: This dataset is stored in separate files for grades 1-4 and it consists of a total of 68 696 unique words (Table 3). Unique word list also includes the frequencies that show an insight into the importance of the words (Table 6).

**Table 3:** Names of files and number of entries for each file in the dataset of Uzbek primary school unique words.

File name	# of entries
unique_words_first_grade.txt	7,973
unique_words_second_grade.txt	14,847
unique_words_third_grade.txt	21,121
Unique_words_fourth_grade.txt	24,755

**Table 1:** Overview of dataset structure.

Dataset Name	Description	# of files	File Names
EDUL.txt	Words from the ``Explanatory Vocabulary of the Uzbek Language''	1	EDUL.txt
Uzbek Primary School Corpus (Grades 1-4)	Textbooks for grades 1-4 approved by the Ministry of Preschool and School Education of Uzbekistan	4	Grade1.txt, Grade2.txt, Grade3.txt, Grade4.txt
New Vocabulary Dataset for Grades 1-4	Newly identified lemma words extracted from the Uzbek Primary School Corpus for each grade level	4	Grade1_Vocabulary.txt, Grade2_Vocabulary.txt, Grade3_Vocabulary.txt, Grade4_Vocabulary.txt,
New words Dataset for Grades 1-4	New words from newly identified lemma words	4	new_words_grade_1.txt, new_words_grade_2.txt, new_words_grade_3.txt, new_words_grade_4.txt
Dataset of Unique Words	Unique words from the Uzbek Primary School Corpus	4	unique_words_first_grade.txt, unique_words_fourth_grade.txt, unique_words_second_grade.txt, unique_words_third_grade.txt
Dataset of Unique Words with Frequencies	Unique words from Uzbek Primary School Corpus with frequencies	4	frequency_unique_grade_1.txt, frequency_unique_grade_2.txt, frequency_unique_grade_3.txt, frequency_unique_grade_4.txt

Based on the above dataset, two datasets were developed:

○ Vocabulary dataset for grades 1-4 of primary school using the method recommended by the authors (Table 4).

**Table 4:** Files and number of entries in the vocabulary dataset for grades 1-4.

File name	# of entries
Grade1_Vocabulary.txt	3,188
Grade2_ Vocabulary.txt	4,630
Grade3_ Vocabulary.txt	5,700
Grade4_ Vocabulary.txt	6,397
○ Dataset of new words that students should know for each academic year (Table 5).

**Table 5:** Dataset of new words introduced to primary school pupils each year.

File name	# of entries
new_words_grade_1.txt	3,188
new_words_grade_2.txt	1,997
new_words_grade_3.txt	1,578
new_words_grade_4.txt	1,356

**Table 6:** An extension of the files from Table 2, but with added frequencies.

File name	# of entries
frequency_unique_grade_1.txt	7,973
frequency_unique_grade_2.txt	14,847
frequency_unique_grade_3.txt	21,121
frequency_unique_grade_4.txt	24,755

**A dataset of unique words for grades 1-4** is created after processing the raw text based on the corpus of the Uzbek primary school. The words in this dataset retain their suffixes because they are taken directly from the raw text without modification.

The dataset of each class is arranged alphabetically and each word appears only once.

Example 1 *The word “haqida” (about) appears 75 times, in the 1^st^ grade unique word dataset but is counted as one unique word.*

Example 2 *The word ``qo'llar'' (hands) appears 100 times in the 3rd grade unique word dataset and is added to the vocabulary dataset as ``qo'l'' (hand) without the suffix ``lar''.*

**The vocabulary dataset for grades 1-4** was established using the method proposed by the authors of the article. Employing the Comparative Lemma Extraction Method, this dataset comprises lemma words. This unique contribution to the education system enables us to address the question: 'How many words should a primary school pupil know each academic year**?**

The creation of the vocabulary makes great opportunities for pedagogues to provide basic knowledge to pupils. Including:

• creation of various didactic materials and software applications for primary school;
• increase pupils vocabulary base;
• teaching pupils to write essays based on different complex grammar rules;
• to teach how to write the words in the vocabulary without mistakes;

In addition, the vocabulary dataset facilitates automated text-processing tasks related to natural language processing (NLP) research.

From the following example, we can see that the vocabulary dataset only includes lemma words.

Example 3 *The word ``ajdod'' (ancestor) appears in various word forms in dataset of Uzbek Primary School Corpus: ``ajdod'' (ancestor), ``ajdodimiz'' (our ancestor), ``ajdodlar'' (ancestors), ``ajdodlardan'' (from ancestors), ``ajdodlarga'' (to ancestors), ``ajdodlari'' (their ancestors), ``ajdodlarimiz'' (our ancestors), ``ajdodlarimizdan'' (from our ancestors), ``ajdodlarimizni'' (our ancestors), ``ajdodlarimizning'' (of our ancestors). However, it is included to the vocabulary dataset as the unique word “ajdod”*

According to the final results of this research, the number of words and new words that each grade pupil should know is as follows:

• First-grader pupils should know 3,188 words, all of which are new words that pupils should know during the school year.
• Second-grade pupils should know 4,630 words, including 1,997 new words.
• Third graders should know 5,700 words, with 1,578 new words derived from them.
• Fourth-grade pupils should know 6,397 words, including 1,356 new words**.**

A dataset of new words that pupils should acquire each school year was generated from “The vocabulary dataset” using custom Python code. The new words for every grade were sorted alphabetically and saved in separate files. This dataset has many advantages in improving the knowledge level of primary school pupils, including:

• Pupils of grades 1-4 will have a source of new words appropriate for their age.
• Pupils can learn the rules of correct writing and reading based on these words.
• By studying the translation of these vocabularies into Russian or English, they can further increase their vocabulary.
• They can learn whether new words have a noun, verb, or adjective form.
• Teachers can use this new set of words to develop dictation exercises, essays, and other types of independent work assignments covering these words throughout the school year.
• Children's writers can write books based on new vocabulary that matches the pupils' intellectual abilities.

### 3.1 Experimental design, materials and methods

The main goal of this study is to determine the number of new words that a primary school pupil should know during each school year. For this, it is necessary to transfer words from school textbooks to lemma form. Since there is no lemmatization tool for the Uzbek language, the comparative lemma detection method was used. Primary school is the main period of learning in a person's life. Having a broad mind and vocabulary during primary school enables mastery of specialized subjects in the future. Creativity, language learning, and the ability to freely express thoughts develop as well. The key is that pupils with sufficient knowledge and vocabulary are confident, and motivated to study, develop, and improve themselves. To achieve all this, educators today must ensure that primary school pupils achieve a high level of vocabulary. So far, no research has determined how many words pupils should know each school year in Uzbek primary schools. Therefore, the findings of this article are crucial for teachers in the education system and for NLP researchers conducting further research on Uzbek language development.

The Uzbek Primary School Corpus (UPSC) is a carefully curated collection of 35 school textbooks written in the Uzbek language, all of which are available online. (https://kitob.uz/ or Android app: Maktab darsliklari | Kitoblar.). This corpus is not only designed to identify new words but also opens up great opportunities for teachers to improve the quality of education and NLP tools.

Several factors motivated the selection of these texts for the corpus:

• Enhancing vocabulary knowledge during the school years.
• Availability of the texts at no cost.
• The reliability of school textbooks, as they are strictly checked for errors.

Some basic data about the corpus:

• name: UPSC;
• total number of words: 208,204;
• number of unique words: 68,696;
• Number of words: 19,915
• New words: 8,119

Due to insufficient availability of software capable of lemmatizing Uzbek language texts, we compiled the ``Explanatory Vocabulary of the Uzbek Language'' (EDUL) and used it for our proposed method. This vocabulary, covering the entire vocabulary of the Uzbek language, has been converted into an ordered list of 29,190 words. By comparing this vocabulary with a dataset of unique words, we created a vocabulary. Thus, we determined the number of new words that primary school pupils should learn during each school year. Details of the algorithm used in this process are provided in the Methods section.

### 3.2 Methods overview

Agglutinative languages, known for their complex morphological structures and heavy use of affixes, pose distinct challenges in natural language processing (NLP). Developing lemma-like vocabularies is essential for applications like machine translation, text mining, and automated text analysis. This study examines the methods and importance of creating lemma-based vocabularies, especially for agglutinative languages such as Uzbek.

Languages like Turkish, Karakalpak, and Uzbek use a variety of suffixes and prefixes to alter word meanings and grammatical roles, resulting in significant word variation and complexity. Accurate syllabification is crucial for NLP. The paper [5] introduces a dual approach to Uzbek syllabification, combining rule-based methods and machine-learning algorithms. The rule-based method addresses syllable division and hyphenation, while the machine learning model is trained on word-syllable mappings, achieving over 99% accuracy. This study offers insights for future research on syllabification in Uzbek and other low-resource Turkic languages. Similarly, a two-level morphological analyzer for Turkish has been influential in further agglutinative language studies [6]. The Comparative Lemma Extraction Method, developed in this research, builds on morphological analysis principles. It involves comparing word forms in a corpus with a reference vocabulary to extract lemma forms. This approach has been applied to various agglutinative languages to standardize vocabulary and improve text processing accuracy. For instance, Finnish research used morphological analysis to create a comprehensive lemma-based vocabulary, significantly enhancing text analysis tasks [7].

The paper focuses on developing a lemmatization algorithm tailored to the Uzbek language. The main objective is to systematically remove word affixes in Uzbek using a finite state machine and accurately identify the lemma, which corresponds to the base form of the word in the vocabulary. The lemmatization process involves using an affix database and part-of-speech patterns specific to Uzbek. It includes defining general rules and categorizing affixes, implementing affix removal through a finite state machine for each affix class, and ultimately determining the lemma form of the word [8]. This paper [9] introduces a machine transliteration tool for the Uzbek language, supporting Cyrillic, official Latin, and the newly announced New Latin scripts. Using rule-based and fine-tuning approaches, it preserves meaning and pronunciation. The tool is available as an open-source Python package and a web-based application with a public API. The paper [10] addresses the lack of linguistic resources for sentiment analysis in low-resource languages by providing the first annotated corpora for Uzbek polarity classification. The authors collected a medium-size manually annotated dataset and a larger automatically translated one, training the first sentiment analysis models for Uzbek with traditional and deep learning techniques, achieving up to 89.56% accuracy. Semantic relatedness between words is a core concept in natural language processing, making semantic evaluation crucial. This paper [11] introduces SimRelUz, a semantic model evaluation dataset for the low-resource Uzbek language. SimRelUz includes over a thousand word pairs, carefully selected based on morphological features, frequency, and semantic relations, and annotated by eleven native Uzbek speakers across various age groups and genders. Special attention was given to rare and out-of-vocabulary words to ensure robust evaluation of semantic models.

### 3.3 A comparative lemma extraction method

We propose a Comparative Lemma Extraction Method (CLEM) to overcome the lack of a powerful lemmatization tool in Uzbek. Our research on a primary school corpus has shown that CLEM works with high accuracy and is recommended for any low-resource language. The Comparative Lemma Extraction Method (CLEM) is a technique used in natural language processing (NLP) and computational linguistics to extract lemma forms from words.

Using the proposed method, each file created from the list of unique words for grades 1-4 is compared with the list of lemma words obtained from the ``Explanatory Vocabulary of the Uzbek language'' dataset. For each unique word in the file, if the EDUL dataset contains a lemma matching the beginning of the unique word, that lemma is extracted and written to a new file in list format. As a result of this method, lemma-based vocabularies with a separate file are created for primary school pupils of grades 1-4. The pseudo-code for detecting lemma words from primary school textbooks based on the CLEM method is outlined in Algorithm 1.

**Algorithm 1:** Lemmata list produced using the CLEM method.

Input (grades);
grades ← [1, 2, 3, 4];
for i = 1 to len(grades) do
begin
unique_words = read_unique_words_file(grades(i));
edul_lemmata = load_edul_lemma_words();
extracted_lemmas = [];
for j = 1 to len(unique_words_file) do
begin
for k = 1 to len(edul_lemma_words) do
begin
if starts_with(unique_words(j), edul_lemma_words(k)) then
extracted_lemmas = extracted_lemmas + edul_lemmata(k);
end;
end;
write_to_file(extracted_lemmas, ``grade_'' + grades(i) + "_lemmas.txt");
end;

As a result, a vocabulary of classes 1-4 was created. Table 7, Table 8, Table 9, Table 10 show part of the list of words for pupils of 1-4 grades:

**Table 7:** Part of Vocabulary List for 1st Grade

Nr.	Word
...	
28.	ahvol
29.	ajdarho
30.	ajdod
...	
3188.	zumrad

**Table 8:** Part of Vocabulary List for 2nd Grade.

Nr.	Word
...	
98.	andisha
99.	andoza
100.	angidrid
...	
4630.	zuvala

**Table 9:** Part of Vocabulary List for 3rd Grade

Nr.	Word
...	
701.	boychechak
702.	boylik
703.	boyqush
5672.	zog'ora
...

**Table 10:** Part of Vocabulary List for 4th Grade.

Nr.	Word
...	
1011.	chizma
1012.	chizmachilik
...	
4249.	ramazon
...	

**The dataset of new words learned each school year** by pupils at each stage lists all new words separately for grades 1-4 that pupils are expected to learn.

The dataset of new words learned each school year by pupils at each stage lists all new words separately for grades 1-4 that pupils are expected to learn. The final result of this article is to determine how many new words a primary school pupil should learn during each school year. The final results are shown in Table 11, Table 12, Table 13, Table 14.

**Table 11:** Part of Dataset of New Vocabulary for 1st Grade. According to our results, this table includes 3188 new words that a 1st-grade pupil should learn during the school year.

Nr.	Word
...	
106.	arslon
107.	arxeolog
...	
3011.	yashnamoq
...

**Table 12:** Part of Dataset of New Vocabulary for 2nd Grade.According to the results of our research, a 2nd grader should learn 1997 new words.

Nr.	Word
1.	adab
...	
101.	baquvvat
...	
1972.	zargarlik
...

**Table 13:** Part of dataset of new vocabulary for 3rd grade. According to our results, a 3rd grader learns 1578 new words during the academic year.

Nr.	Word
1.	a'lam
...	
205.	chambarak
...	
1546.	zahmatkash
...

**Table 14:** Part of dataset of new vocabulary for 4th grade. According to the final results, a 4th-grade pupil learns a total of 1356 new words that were not encountered in the previous school years.

Nr.	Word
...	
63.	avtomobilsozlik
...	
446.	jannatmakon
...	
1356.	zora

Table 11 shows part of the table of new words that a 1st-grade pupil should learn during the school year. According to the results, we can say that a 1st-grade pupil should learn a total of 3188 new words during the school year.

Table 12 shows an excerpt of new words that 2nd graders should know. According to the results of our research, in addition to the new words that 2nd graders learned in 1st grade, there are 1997 more new words to learn, and the number of new words learned in the first and second grades reaches 5195.

Table 13 shows a selection of new words that 3rd graders should know. According to our results, a 3rd grader learns 1578 new words during the academic year, and the number of new words learned during the 3 academic years reaches 6,773.

Finally, Table 14 shows some of the words that a 4th-grade pupil should learn during the school year. According to the final results, in the 4th grade, the pupil learns a total of 1,356 new words that he did not encounter in the previous school years, and the total number of new words learned during the 1-4 stages of primary education reaches 8,119. Table 14 presents the final results of this research (Table 15).

**Table 15:** Overall Statistics about Dataset of New Vocabulary for 1st-4th Grades. A 1st grader should know 3188 new words. A 2nd grader learns 1997 new words during the school year. Adding the new words from the first and second grades, the vocabulary reaches 5195. A 3rd grader learns 1578 new words during the school year, and together with the new words from the first and second grades, their vocabulary reaches 6773 words. Finally, according to the final results, a 4th-grade pupil learns 1356 new words during the school year. Including the new words learned in the 1st, 2nd, and 3rd grades, their vocabulary totals 8119 words. Thus, the number of new words known by a pupil who has completed the 4th grade of primary school is 8119 words.

Grade	Number of New Words Acquired During School Year	Total Number of Words Pupils Should Know
1st	3,188	3,188
2nd	1,997	5,195
3rd	1,578	6,773
4th	1,356	8,119

## Limitations

The scope of the paper is to determine how many new words primary school students should acquire in each academic year. The frequency of lemma words in the dataset is not present although we are aware that this would extend the usefullness of the presented data.

## Ethics statement

The data-gathering process did not involve the use of human subjects. The only possibly problematic ethical aspect comes from the possible privacy concerns of the gathered corpus and consequently the extracted word lists. All the gathered texts are from public school materials that were priory checked and recognized as without any ethical concerns.

Regarding the data redistribution policies to be complied with, written consent was obtained from the book.uz platform owners with, which is The Republican Center for Education under the Ministry of Public Education of the Republic of Uzbekistan, on April 7, 2022, with the registration number 01/11-01/7-418.

## Credit Author Statement

**Khabibulla Madatov:** Conceptualization, Software, Investigation, Supervision, Writing – Review & Editing. **Sapura Sattarova:** Software, Investigation, Writing – Review & Editing, **Jernej Vičič:** Conceptualization, Investigation, Writing – Review & Editing, Data Curation.

## Data Availability

ZenodoDataset of Vocabulary in Uzbek Primary Education (Original data).

## References

[1] Madatov K., Bekchanov S., Vičič J. (2023). Proceedings of The 10th Language & Technology Conference.

[2] Madatov K., Bekchanov S., Vičič J. Dataset of stopwords extracted from Uzbek texts //Data in Brief. –2022. – Т. 43. – С. 108351. 10.1016/j.dib.2022.108351 PMC9194838 35712366

[3] Madatov K., Bekchanov S., Vičič J. (2022). CEUR Workshop Proceedings.

[4] Madatov K., Bekchanov S., Vičič J. (2023). Automatic detection of stop words for texts in the Uzbek language. Informatica (Slovenia).

[5] Salaev U.I., Kuriyozov E.R., Matlatipov G.R. (2023). 2023 IEEE XVI International Scientific and Technical Conference Actual Problems of Electronic Instrument Engineering (APEIE).

[6] Oflazer K. (1994). Two-level description of Turkish morphology. Literary Linguistic Comput..

[7] Pirinen T.A. (2015). Development and use of computational morphology of Finnish in the open source and open science era: Notes on experiences with Omorfi development. Linguistic Issues Lang. Technol..

[8] Sharipov M., Sobirov O. Development of a rule-based lemmatization algorithm through Finite State Machine for Uzbek language //arXiv preprint arXiv:2210.16006. –2022.

[9] Salaev U., Kuriyozov E., Gómez-Rodríguez C. (2022). CEUR Workshop Proceedings.

[10] Kuriyozov E., Matlatipov S., Alonso M.A. (2022). Gomez-Rodr´ ´ıguez C., Construction and evaluation of sentiment datasets for low-resource languages: The case of uzbek. in proceedings of the language and technology conference.

[11] Salaev U., Kuriyozov E., Gómez-Rodríguez C. (2022). 1st Annual Meeting of the ELRA/ISCA Special Interest Group on Under-Resourced Languages SIGUL 2022 - held in conjunction with the International Conference on Language Resources and Evaluation LREC 2022 - Proceedings.

[12] S.G‘.Matlatipov, X.A.Madatov, G‘.R.Matlatipov, A.D.O‘razbayev, M.K.Raximboyev, I.D.Avezmatov, U.U.Babajanov, L.U.Kurbanova, D.J.Xujamov, D.Q.Matjumayeva. “O‘zbek tilining statistik electron lug‘at” EXM dasturi uchun guvohnoma, № DGU09919, 09.12.2020;

